# An examination of the direct and indirect effect of self-objectification and disordered eating

**DOI:** 10.1192/j.eurpsy.2021.1868

**Published:** 2021-08-13

**Authors:** S. Giles, J. Rabinowicz, C. Raux, M. Fuller-Tyszkiewicz, I. Krug

**Affiliations:** 1 Melbourne School Of Psychological Sciences, The University of Melbourne, Parkville, Australia; 2 Psychology, Institute of Social Neurosciences Psychology College, Ivanhoe, Australia; 3 Centre For Social And Early Emotional Development, Deakin University, Burwood, Australia

**Keywords:** eating disorders, Self-objectification, disordered eating, ecological momentary assessment

## Abstract

**Introduction:**

Objectification theory argues that self-objectification confers risk for disordered eating (DE) both directly, and indirectly through a cascade of negative psychological consequences (e.g. low mood and self-conscious body monitoring). Robust cross-sectional evidence supports these relationships. However, these cross-sectional studies do not provide evidence for the complex intraindividual psychological processes outlined in objectification theory which purportedly contribute to DE.

**Objectives:**

Using an ecological momentary assessment design, the current study investigated the direct within-person effect between state self-objectification and DE and examined the indirect within-person effect of negative mood and body comparisons, on the relationship between state self-objectification and DE.

**Methods:**

Two-hundred female participants (M=20.43 years, SD=4.60) downloaded a smartphone app which assessed momentary experiences of self-objectification, mood, body comparisons, and DE six times per day at random intervals for seven days.

**Results:**

Indicated that self-objectification significantly predicted DE behaviours [95% CI 0.01, 0.03] and body comparisons [95% CI 0.32, 0.41]. However, the indirect effect of body comparisons on the relationship between state self-objectification and DE was not significant [95% CI -0.01, 0.00]. In the second mediation model, self-objectification significantly predicted DE behaviours [95% CI 0.01, 0.03], but did not significantly predict mood [95% CI -0.06, 0.03]. Similarly, the indirect effect of mood on the relationship between state self-objectification and DE was not significant [95% CI -0.00, 0.00].

**Conclusions:**

These results enhance our understanding of objectification theory and suggest that self-objectification confers risk to DE directly. However, our findings do not support the indirect effect of self-objectification on DE through low mood or body comparisons.

**Disclosure:**

No significant relationships.

